# Recommended Tool Compounds: Application of YM-254890 and FR900359 to Interrogate Gα_q/11_-Mediated Signaling Pathways

**DOI:** 10.1021/acsptsci.3c00214

**Published:** 2023-11-10

**Authors:** Jan Hendrik Voss

**Affiliations:** Department of Physiology and Pharmacology, Section of Receptor Biology and Signaling, Karolinska Institutet, S-171 65 Stockholm, Sweden

**Keywords:** FR900359, G protein, tool compound, G protein-coupled receptor, review, YM-254890

## Abstract

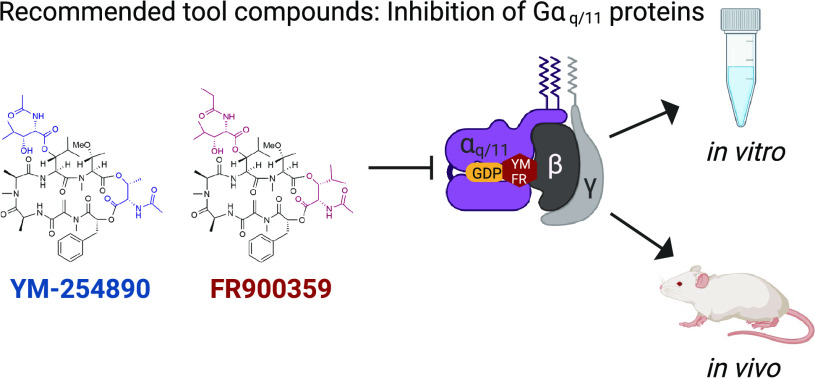

The macrocyclic depsipeptides YM-254890 (YM) and FR900359 (FR) are natural products, which inhibit heterotrimeric Gα_q/11_ proteins with high potency and outstanding selectivity. Historically, pharmacological modulation of Gα proteins was only achieved by treatment with pertussis toxin and cholera toxin, whose application can be tedious and is restricted to the inhibition of Gα_i/o_ proteins and activation of Gα_s_ proteins, respectively. The breakthrough discovery and characterization of YM and FR rendered the closely related Gα_q_, Gα_11_, and Gα_14_ proteins amenable to pharmacological inhibition, and since then, both compounds have become widely used in molecular pharmacology and were also proven to be efficacious in animal models of disease. In the past years, both YM and FR were thoroughly characterized and have substantially contributed to an improved understanding of Gα_q/11_ signaling on a molecular and cellular level. Yet, the possibilities to interrogate Gα_q/11_ signaling in complex systems have only been exploited in a very limited number of studies, whose promising initial results warrant further application of YM and FR in basic and translational research. As both compounds have become commercially available as of late, this review focuses on their application in cell-based assays and in vivo systems, highlighting their qualities as tool compounds and providing instructions for their use.

G protein-coupled receptors (GPCRs) are the largest family of membrane proteins in the human genome, comprising approximately 800 members.^1^ GPCRs are the subject of many pharmacological studies, and drug development targeting GPCRs has been tremendously successful due to the modular nature of GPCRs in a complex signaling cascades, their well-accessible binding site, and their recognition of a wide range of chemotypes, e.g., small molecules, peptides, proteins, and lipids.^2^ The most immediate intracellular effector proteins of GPCRs are heterotrimeric guanine-nucleotide binding proteins (G proteins), which relay signaling from activated receptors to intracellular signaling pathways.^3^ G proteins are composed of a Gα, Gβ, and Gγ protein subunit, and are located on the inner leaflet of the plasma membrane. Here, the Gα protein is the key factor determining the downstream signaling pathway(s) initiated by an active-state GPCR.^4^ Notably, free Gβγ subunits can activate distinct effector proteins, yet the role of Gβγ effector specificity has only partially been uncovered.^5,6^ GPCR signaling converges on the level of G proteins. In total, the human genome encodes for only 16 Gα protein subunits, grouped by sequence similarity into the four subfamilies Gα_s_, Gα_i/o_, Gα_q/11_, and Gα_12/13_ (Figure 1), as well as 5 Gβ subunits, and 12 Gγ subunits.^7^

**Figure 1:**
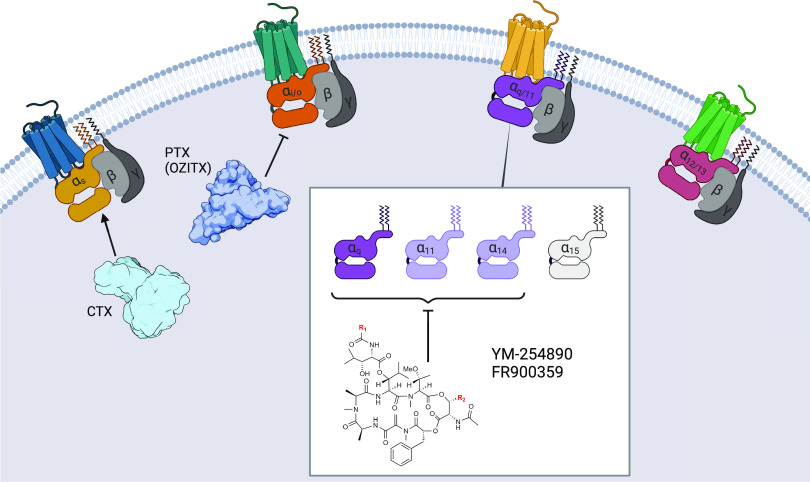
Pharmacological modulation of Gα protein subunits. Active state GPCRs promote guanine nucleotide exchange in Gα protein subunits, which then initiate downstream signaling cascades. Gα_s_ proteins are ADP-ribosylated by cholera toxin (CTX), leading to a loss of catalytic activity in the Gα_s_ protein, thereby keeping the Gα_s_ protein in its active GTP-bound state. Gα_i/o_ proteins (except for Gα_*z*_) are ADP-ribosylated in their C-termini, blocking interaction with the GPCR, which inactivates the G protein. The Gα_q/11_ family members Gα_q_, Gα_11_, and Gα_14_ are inactivated by the macrocyclic compounds YM and FR, which prevent nucleotide exchange from GDP to GTP upon receptor activation. To date, Gα_12/13_ proteins are not amenable to pharmacological modulation. YM: R_1_ = CH_3_, R_2_ = CH_3_; FR: R_1_ = C_2_H_5_, R_2_ = C(CH_3_)_2_.

A detailed investigation of GPCR-mediated signaling networks can be cumbersome, as most GPCRs do not exclusively activate a single Gα protein or Gα protein subfamily, but rather two or more Gα protein families, recruit β-arrestins, and display complex spatiotemporal signaling patterns.^8−10^ While several recently developed bioluminescence energy resonance transfer (BRET) biosensors facilitate cellular studies of individual GPCR-Gα interactions, this setup relies on the overexpression of artificial protein constructs in cultured cells.^9,11,12^ To study the downstream signaling of specific GPCRs in native cells and tissues or whole organisms, potent tool compounds selectively inhibiting individual Gα proteins or subfamilies are highly desirable. However, Gα proteins are much less amenable to pharmacological modulation than their activating receptors. While several compounds, mostly of peptidic nature, have been proposed to act as Gα protein modulators, most of them are not used routinely as tool compounds. This points to issues regarding potency, efficacy, cell-permeability, synthesizability, and subtype selectivity^7^

Historically, exclusively the bacterial toxins cholera toxin (CTX; activator of the Gα_s_ protein) and pertussis toxin (PTX; inhibitor of the Gα_i/o_ protein family with exception of Gα_*z*_) were used as pharmacological modulators of Gα proteins in basic research (Figure 1).^13−15^ In the early days of G protein research and GPCR pharmacology, CTX and PTX played a major role in identifying the contribution of Gα_s_ or Gα_i/o_ proteins to a given biochemical event, but their macromolecular structure, their toxicity, and their limited coverage of Gα proteins have drawbacks for their everyday use. The complex pathological mechanism of CTX, reviewed in ref (15), results in adenosine diphosphate (ADP)-ribosylation of the Gα_s_ protein nucleotide binding site, which leads to a loss of its catalytic activity by keeping Gα_s_ in a guanosine triphosphate (GTP)-bound state, subsequently uncoupling cAMP production from receptor activation. PTX inhibits Gα_i/o_ proteins (with exception of the Gα_*z*_ protein) by ADP-ribosylation of a C-terminal cysteine, thereby preventing the recognition of active-state GPCRs and subsequent receptor-mediated nucleotide exchange in ADP-ribosylated Gα proteins. Recently, a novel PTX-like protein, termed OZITX, was engineered to ADP-ribosylate a Gα_i/o_-family wide conserved C-terminal asparagine residue and is thus capable of targeting Gα_*z*_ proteins.^16^

In the past decade, the macrocyclic depsipeptides YM-254890 (YM) and FR900359 (FR), also known by the name UBO-QIC, have become widely used tool compounds in molecular pharmacology as specific inhibitors of the Gα_q/11_ protein family (Figure 1).^17^ In a cellular context, activation of Gα_q/11_ proteins induces phospholipase C-β (PLC-β)-mediated production of the second messenger molecules diacylglycerol (DAG) and inositol trisphosphate (IP_3_) and subsequent liberation of Ca^2+^ from intracellular storages.^18^ Further signaling pathways triggered by Gα_q/11_ protein activation include (but are not limited to) several pro-mitogenic pathways, such as extracellular signal regulated kinase (ERK) phosphorylation, Akt phosphorylation, RhoA stimulation, and Hippo/YAP signaling, associating Gα_q/11_ protein activation with cancer malignancy.^18,19^ Accordingly, mutational activation of Gα_q/11_ proteins was found to be causative for uveal melanoma, the most prevalent type of eye cancer in humans.^19−21^

Both YM and FR act as guanine nucleotide dissociation inhibitors (GDI), i.e., they prevent the dissociation of guanosine diphosphate (GDP) from the inactive Gα protein triggered by interaction with an active GPCR, and therefore arrest the Gα protein in its inactive state, irresponsive to stimuli from active GPCRs.^22,23^ Out of the four members of the Gα_q/11_ protein family, YM and FR engage three of them (Gα_q_, Gα_11_, Gα_14_) with high affinity, sparing the evolutionarily more distant Gα_15/16_ proteins (Gα_15_ and Gα_16_ denote the human and mouse paralog, respectively).^22,24^ Following the recent commercialization of FR, this review, as a part of the review series on recommended tool compounds published in *ACS Pharmacology and Translational Science*,^25^ aims to deliver an in-depth summary focused on the pharmacology and application of YM and FR.

## Chemical Structure and Biosynthesis

**Figure 2:**
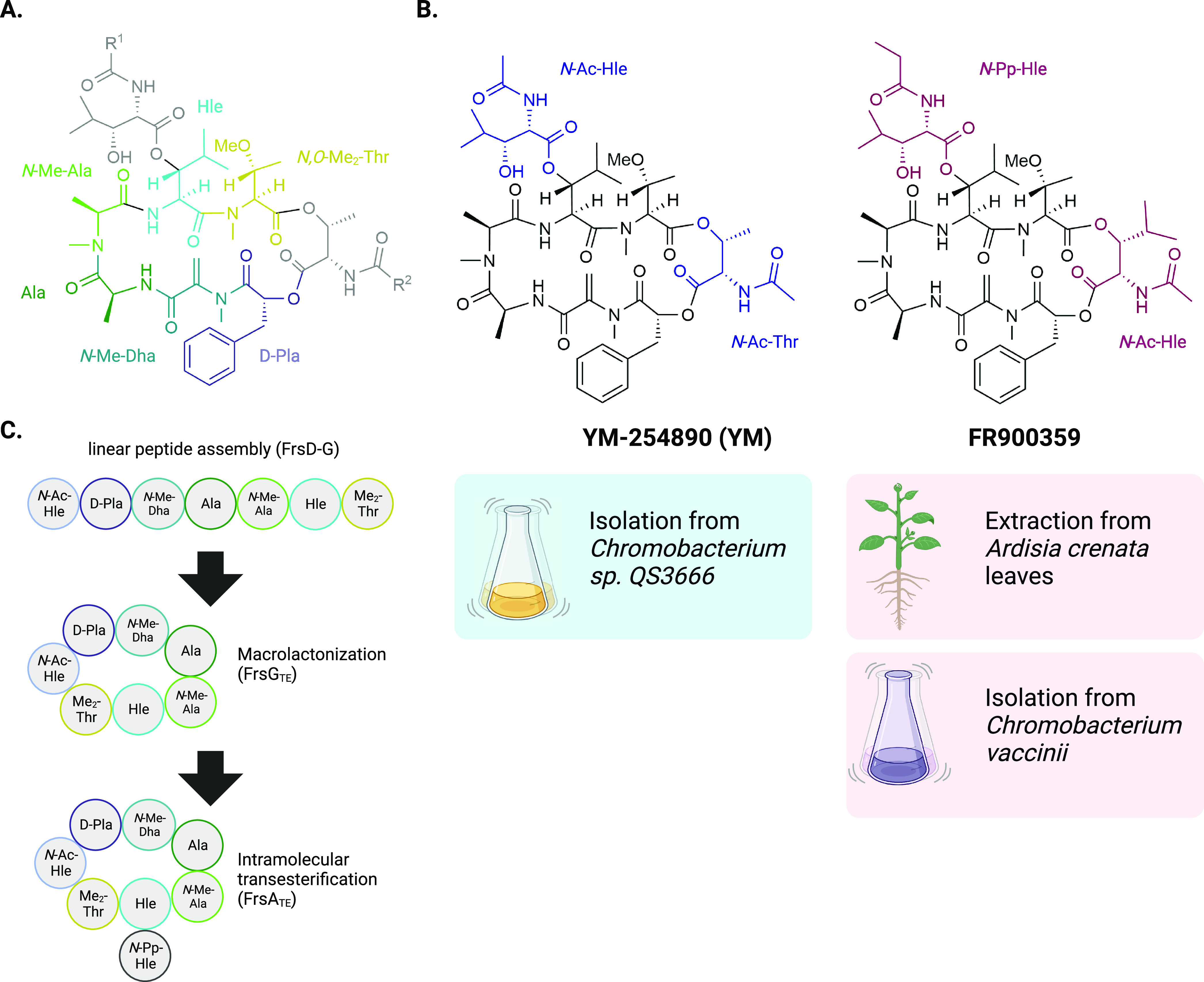
Chemical structure and biosynthesis of YM and FR. (A) Common chemical structure of YM and FR (YM: R^1^ = CH_3_, R^2^ = CH_3_; FR: R^1^ = C_2_H_5_, R^2^ = C(CH_3_)_2_). Shared residues forming the macrocyclic backbone are highlighted in different colors. d-Pla, d-phenyllactic acid; *N*-Me-Dha, *N*-methyl-dehydroalanine; Ala, alanine; *N*-Me-Ala, *N*-methyl-alanine; Hle, hydroxyleucine; *N*,*O*-Me_2_-Thr, *N*,*O*-dimethyl-threonine. (B) Chemical structures of YM (left) and FR (right). Unique residues are highlighted in blue (for YM) and purple (for FR), respectively. *N*-Ac-Hle, *N*-acetyl-hydroxyleucine; *N*-Ac-Thr, *N*-acetyl-threonine; *N*-Pp-Hle, *N*-propionyl-hydroxyleucine. Procedures to isolate each natural product are listed below the respective chemical structure. (C) Schematic depiction of the FR biosynthetic pathway, as postulated by Hermes et al.^29^

YM and FR are natural products of bacterial origin sharing a nearly identical, complex structure, which differs only in two residues (Figure 2A+B). Their macrocyclic backbone is composed out of seven building blocks (d-phenyllactic acid (d-Pla), *N*-methyl-dehydroalanine (*N*-Me-Dha), alanine (Ala), *N*-methyl-alanine (*N*-Me-Ala), hydroxyleucine (Hle), *N–O*-dimethyl-threonine (*N*,*O*-Me_2_-Thr), and *N*-acetyl-hydroxyleucine (*N*-Ac-Hle; *N*-acetyl-threonine (*N*-Ac-Thr) in YM, see Figure 2B). A further hydroxyleucine side chain, which is *N*-acetylated in case of YM (*N*-Ac-Hle) and *N*-propionylated (*N*-Pp-Hle) in case of FR, is attached to the hydroxyl group of the core hydroxyleucine residue.

YM was isolated from *Chromobacterium sp.* QS3666, a bacterial strain discovered in a soil sample from Tokyo, Japan.^26^ The biosynthetic process yielding YM is unknown, as this bacterial strain was never made publicly available and no related information about the biosynthetic gene cluster (BGC) producing YM can be retrieved from the original publication. FR had first been isolated from the leaves of the plant *Ardisia crenata* in 1988,^27^ but only later in 2015, it was pharmacologically characterized as a potent and specific Gα_q/11_ protein inhibitor.^22^ As FR potently inhibits insect Gα_q_ proteins, it protects the plant from predatory insect larvae.^28^ Subsequently, a large effort has been put into the elucidation of FR biosynthesis, whose biosynthetic pathways will be outlined in brief here. For a more comprehensive review focusing on the discovery and biosynthesis of FR, see Hermes et al.^29^

FR is naturally produced by the bacterium *Candidatus burkholderia crenata*, which is located in leaf nodules of *A. crenata*.^28,30^ This obligate endosymbiont harbors a BGC on a plasmid encoding for eight genes, termed frsA-H. In the proposed synthesis pathway, a nonribosomal protein synthetase composed of the enzymes FrsD-G forms a seven-residue linear peptide chain which is cyclized by the FrsG transesterification domain. In a final step, the propionylated β-hydroxyleucine side chain is attached to the free hydroxyl group of the FR-core molecule by the FrsA transesterification domain (Figure 2C).^29,31^ A highly similar BGC was discovered in *Chromobacterium vaccinii*, which, in contrast to *Cand. burkholderia crenata*, is a cultivable producer of FR, yielding approximately 2.5 mg FR per liter of bacterial culture.^31,32^

The natural producers of both YM and FR do not exclusively synthesize the respective compounds, but also generate several related side products harboring minor chemical modifications.^31−34^ Several of these compounds, e.g., FR-1/2 or YM-254891/2, apparently retain the pharmacological properties of the parent compounds, yet already subtle structural deviations were shown to have a profound impact on binding kinetics due to the complex Gα_q/11_ inhibitor pharmacophore.^33−35^ More profound chemical differences, such as truncation of the branched *N*-Ac/Pp-Hle side chain, resulted in a large decrease in affinity.^31,33^ Intriguingly, natural derivatives of YM and FR contained similar modifications relative to the original compound, such modifications or truncation of the side chain, hinting at mechanistically similar biosynthetic pathways of YM and FR. Furthermore, another analogue of YM and FR, Sameuramide A, has been isolated from a marine tunicate,^36^ which is chemically identical to the FR derivative FR-3 (relative to FR, FR-3, and Sameuramide A contain a *N*-Pp-Hle instead of an *N*-Ac-Hle residue in its macrocyclic core). While—similar to YM—information about the BGC yielding Sameuramide A is not available, it is striking that nearly identical compounds with high affinity toward Gα_q/11_ proteins have apparently evolved on at least three occasions.

In addition to the isolation of the compounds from natural sources, the total synthesis of YM and FR was first reported in 2016.^37^ The synthesis approach allowed for a versatile exchange of several building blocks, which led to the generation of several novel YM analogues.^37−40^ In an accompanying structure–activity relationship, none of the generated molecules displayed an increased inhibitory potency in an inositol monophosphate (IP_1_) accumulation assay readout.^37,41^ Exploitation of this synthesis route in the future may generate diverse derivatives, which could display affinities toward other, currently undrugged Gα proteins, in particular the most closely related Gα_15_ protein.

## Chemical and Pharmacokinetic Properties

As the chemical structures of YM and FR are very closely related, it would be intuitive for their chemical and pharmacokinetic properties to resemble each other closely. A direct comparison, however, detected in part substantial differences between both compounds.^42^ YM and FR display sufficient solubility in water for all biological applications displaying a similar solubility product of 189 μM for FR and 88 μM for YM, and a calculated log *P* value of 1.86 and 1.37, respectively.^42^ The solubility in ethanol and DMSO is high, and both compounds can be stored as a powder or in a 1 mM DMSO stock at 4 °C for longer periods of time without notable loss of activity. Chemical stability was found to be very high in simulated gastric fluid and mildly alkaline solution (pH 9), but impaired under strongly alkaline conditions (pH 11). Here, YM decomposed fully and rapidly, whereas only about 25% of FR degraded within a period of 4 h. FR-core, a biosynthesis intermediate lacking the *N*-Pp-Hle side chain, was identified as a decay product under alkaline conditions.^42^ Interestingly, both depsipeptides formed an isomer with equal mass under all conditions, which was identified in a later study as a biologically inactive degradation product that had undergone macrocyclic ring cleavage (between the backbone *N*,*O*-Me_2_-Thr and *N*-Ac-Hle residue of FR/*N*-Ac-r residue of YM) and dehydration (at the *N*-Ac-Hle residue of FR/*N*-Ac-r residue of YM).^43^

An in vitro assessment of YM and FR’s pharmacokinetic properties yielded mostly similar results for both compounds. Their bioavailability was predicted to be low in an intestinal absorption model (Caco2 permeation assay) with the basal-apical transportation rate being greater than the apical-basal transportation rate. The exceptionally high basal-apical transport rate suggests that both compounds are substrates of efflux transporters, such as P-glycoproteins. Given the direction of transport for these compounds, the study postulates a low oral bioavailability and low penetration into the central nervous system. In plasma and lung tissue, YM and FR were found to be relatively stable (<10% decay after 4 h). In contrast, both compounds were metabolized rather quickly in liver microsomes, displaying half-lives of 27 min for YM and a far shorter half-life of 8 min for FR, yet the metabolites and the metabolic pathways remain unknown. Both compounds did not inhibit the investigated drug-metabolizing cytochrome P450 (CYP) enzymes at therapeutically relevant concentrations. At excessive concentrations of 10 μM, YM and FR inhibited the CYP3A4 enzyme by slightly above 50%, but none of the other tested enzymes.^42^

Furthermore, in vivo distribution of both compounds was assessed in mouse, following 7 days of intratracheal application (5 μg YM and FR per animal and day). Organs of interest were homogenated and extracted with methanol, followed by a LC-MS/MS analysis protocol to quantify the levels of YM and FR in the respective organs.^44^ The highest levels of both compounds were detected in the kidney and in the lung. A high level of YM was also found in the liver. FR was not found here, which is likely due to its fast liver metabolization. Upon 21 days of intratracheal application (2.5 μg YM and FR, twice daily), near-exclusive drug accumulation in the lung was observed.^42,45^ In agreement with previously mentioned absorption model data classifying YM and FR as P-glycoprotein substrates, neither compound was detected in mouse brain tissue after intratracheal application.^42^ After a one-time oral application of 0.2 mg FR, the compound could be recovered from several organ extracts, implying that FR is at least to some degree orally bioavailable. After oral application, the highest concentrations of FR were recovered from the gut and remarkably also from the eye, with low compound levels detected in liver, kidney, lung, heart, and fatty tissue.^44^ As tissue distribution was addressed by using LC-MS/MS, metabolites, or other chemically modified derivatives of YM and FR were not quantified from homogenized organ extracts.

In summary, YM and FR do not fulfill the criteria for drug-like molecules in the classical sense defined by Lipinski’s rule of five due to their high molecular weight.^46^ Reflective of that, the oral bioavailability of both compounds is predicted to be low, which is the case for 61% of FDA-approved macrocyclic drugs.^47^ However, when applied by other routes, e.g., intratracheal or intraperitoneal application, YM and FR displayed sufficiently good stability in plasma and lung tissue, no excessive undesired tissue accumulation, and no CYP enzyme inhibition at relevant concentrations, making them generally suitable for in vivo application. A caveat for their in vivo use is the excessive and, especially for FR, quick metabolization yielding unidentified molecules of unknown biological activity.

## Molecular Mechanism of Gα_q_ Protein Inhibition by YM and FR

**Figure 3:**
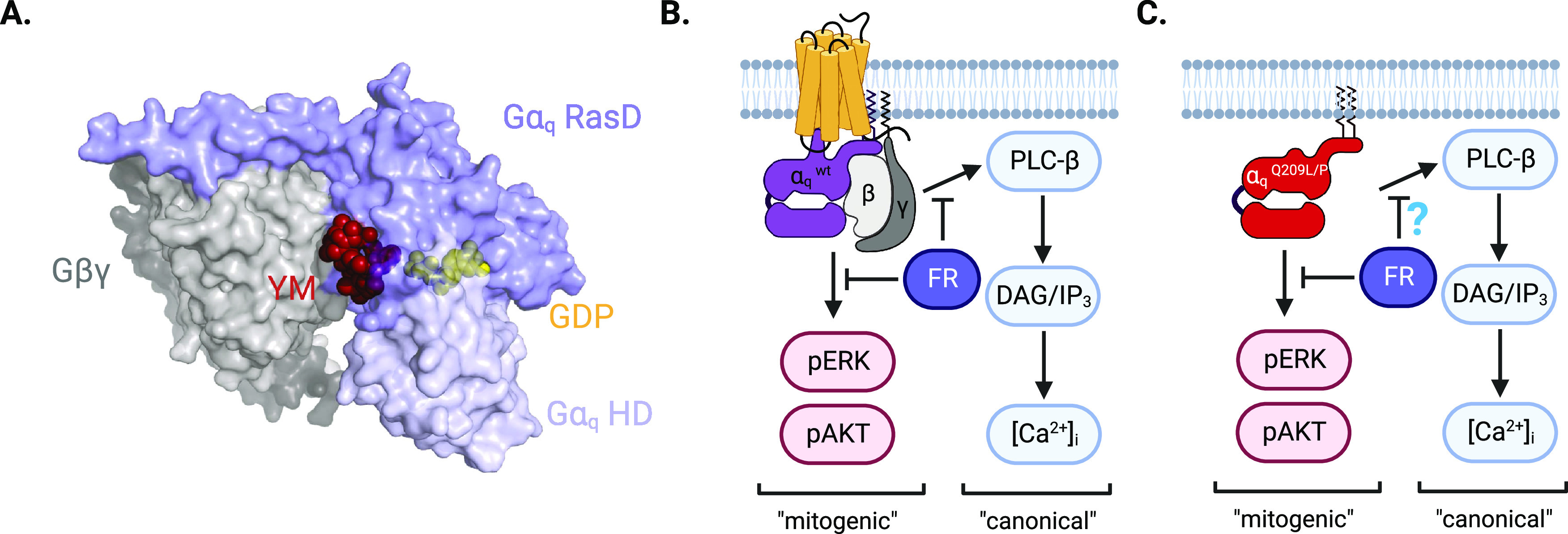
Binding mode and mechanism of action of Gα_q/11_ inhibitors. (A) Surface representation of the heterotrimeric Gα_q_β_1_γ_2_ protein in complex with YM (red) and GDP (yellow) (PDB 3AH8(23)), showing binding of YM to a site located at the linker I/switch I region between the Gα_q_ Ras-like domain (RasD, blue) and its helical domain (HD, light blue) near the interface with the Gβ subunit (gray). (B) Binding of FR to the wild-type (wt) Gα_q_ protein inhibits G protein-dependent signaling in readouts covering different signaling cascades, including mitogenic signaling pathways (exemplified by ERK and Akt phosphorylation, red) and canonical, PLC-β-mediated pathways (blue). (C) Binding of FR to constitutively GTP-bound, receptor-uncoupled Gα_q_^Q209L/P^ preferentially silences mitogenic signaling over PLC-β dependent signaling.

Both YM and FR were characterized as GDIs at Gα_q_, Gα_11_, and Gα_14_ proteins. The mechanism of action has been confirmed for both YM and FR by [^3^H]GDP-dissociation experiments with purified Gα_q_ proteins in two independent sets of experiments.^22,23^ A crystal structure of YM in complex with the heterotrimeric, GDP-bound Gα_q_β_1_γ_2_ protein complex (2.9 Å resolution) demonstrated that the compound binds to the linker I/switch I area of the Gα_q_ protein. This area connects the protein’s Ras-like domain to its helical domain (see Figure 3A), and binding of YM and FR prevents domain separation, which is a prerequisite for nucleotide exchange—the rate-limiting step in Gα protein activation.^23,48^ In this crystal structure, direct interactions with the nearby Gβ subunit were not observed, however, the electron density of the ligand in this structure is in part ambivalent.^49^ In a recent preprint describing high-resolution crystal structures of YM and FR in complex with G_11_ (1.7 and 1.43 Å resolution, respectively), molecular interactions between the N-Ac/Pp-Hle side chain of Gα_q/11_ inhibitors and the Gβ residue R96 were observed. These structures and the supporting functional data suggest an additional mechanism of action for these compounds acting also as “molecular adhesives”, which stabilize a heterotrimeric G protein complex by linking the Gα and Gβγ subunits, thereby preventing molecular recognition of Gα_q/11_ effector proteins.^71^

YM and FR bind Gα_q_ proteins with high affinity and broadly inhibit Gα_q/11_ activity as shown in several signaling readouts (Figure 3B). Radioligand binding experiments with probes derived from YM and FR by catalytic hydrogenation of the exocyclic double bond reported p*K*_D_ values of 7.96 and 8.45 for YM- and FR-derived radiotracers, respectively, in saturation binding assays using human platelet membrane preparations.^24^ Notably, binding of radiolabeled Gα_q_ inhibitors is not allosterically modulated by salts, lipids, GPCR agonists, and nucleotides.^24^A direct comparison of binding parameters is provided in Table 1.

**Table 1:** Binding Constants and Biological Activity of YM and FR in Multiple Assay Systems

binding constants	YM	FR	reference
apparent affinity, saturation binding of derived radioligand, human platelet membranes, 37 °C (*p*K_D_)	7.96	8.45	Kuschak et al.^24^
apparent affinity, pseudohomologous competition binding between radioligand and unlabeled parent compound, human platelet membranes, 37 °C (*p*K_i_)	8.20	8.39	Kuschak et al.^24^
association half-life of derived radioligand, human platelet membranes, 37 °C (min)	3.6	6.7	Kuschak et al.^24^
dissociation half-life of derived radioligand, human platelet membranes, 37 °C (min)	3.8	92	Kuschak et al.^24^
dissociation half-life of parent compound, HEK293 cell membrane, 37 °C (min)	40	323	Voss et al.^35^

biological activity	YM	FR	reference
inhibition of calcium mobilization, HEK293-Gα_q_ cells (*p*IC_50_)	8.09	8.20	Voss et al.^50^
inhibition of carbachol-induced IP_1_ accumulation, CHO-M_1_R cells (*p*IC_50_)	7.03	7.49	Xiong et al.^37^
inhibition of ADP-induced platelet aggregation (IC_50_, μM)	0.26	n.d.	Taniguchi et al.^26,51^
label-free dynamic mass redistribution, stimulated by an EC_80_ conc. of carbachol (*p*IC_50_)	6.3	6.3	Malfacini et al.^52^

Kinetic experiments using these radiotracers unveiled a huge discrepancy in unbinding kinetics between the fast dissociating YM-derived radiotracer (dissociation half-life: 3.8 min) and the slow-dissociating FR-derived radiotracer (dissociation half-life: 92 min), consistent with previous reports claiming that FR-, but not YM-mediated inhibition of Gα_q_ proteins, was washout-resistant.^22,24^ The molecular basis for pseudoirreversible binding of FR was pinpointed to be a stronger lipophilic interaction between the isopropyl group exclusively present in FR and the neighboring amino acids of the Gα_q_ binding site.^24,50^ In a different study, establishing a structure–affinity and structure–kinetics relationship of unlabeled YM, FR, and their derivatives, an even longer dissociation half-life was determined for the parent, non-hydrogenated compound FR (323 min) using a competition association binding setup. Interestingly, the dissociation half-life of YM was also found to be far longer than that of its radiolabeled derivative (40 vs 3.8 min).^35^

Structure–activity relationships, structure–kinetics relationships, and mutagenesis experiments of the Gα_q_ inhibitor binding site found that even minor modifications to the ligand–protein interaction all result in a major decrease of potency, affinity, and residence time, suggesting that major parts of YM and FR (and vice versa, of the Gα_q_ inhibitor binding site) contribute to high-affinity ligand binding.^35,37,50,53^

Initial binding studies and functional characterization demonstrated a high degree of selectivity toward Gα_q_, Gα_11_, and Gα_14_ proteins (which share a fully conserved inhibitor binding site) over the closest paralogue, Gα_15_ (five residue exchanges in the binding site), and all other Gα protein families.^22,24,52^ Gα_15_ inhibition by FR is theoretically possible, but requires high inhibitor concentrations exceeding 1 μM, while YM could not inhibit the Gα_15_ protein at all in the tested concentration range.^52^ Two independent reports have claimed putative off-target action of YM and FR, i.e., not mediated by inhibition of Gα_q/11_ proteins; in one case it was hypothesized that the observed effects are caused by blockade of Gβγ activity following Gα_i/o_ activation,^54^ in the other case by broad-spectrum inhibition of Gα_q/11_ and Gα_s_ proteins and pathway-selective inhibition of Gα_i/o_-mediated signaling.^55^ Making use of an FR-resistant Gα_q_ protein mutant, Patt et al. demonstrated that even at high concentrations, YM and FR did not display off-target effects in a plethora of readouts and any biological effect required the presence of wild-type Gα_q_ proteins, inferring that the previously observed effects can be attributed to Gα_q/11_ inhibition of YM and FR.^56^

A further intriguing aspect of the mode-of-action of FR has come from its application to GTPase-deficient Gα_q_ and Gα_11_ proteins bearing the Q209L/P mutations, which is causative for a notable fraction of uveal melanoma cases in humans.^21^ Gα_q_^Q209L/P^ proteins are constitutively bound to GTP and are therefore active irrespective of receptor input. On the basis of its classification as a GDI, it would be expected that FR cannot inhibit these mutant Gα_q_ proteins. Unsuspectedly, FR (but so far not YM) was found to preferentially inhibit the mitogenic ERK and AKT signaling pathways in cell-based assays without attenuating canonical PLC-β-dependent signaling (measured by IP_1_ accumulation assays) in HCmel12 cells (Figure 3C).^19^ This finding could not be reproduced in several other Gα_q_^Q209L/P^-expressing cell lines, leaving the inhibitory mechanism of FR on PLC-mediated signaling downstream of Gα_q_^Q209L^ incompletely understood at present.^19^ It may be possible that elevated IP_1_ levels in FR-unresponsive cells lines are mediated by high Gα_16_ activity.

Inhibition of mitogenic Gα_q/11_ signaling by application of FR to models expressing Gα_q_^Q209L/P^ mutants has been demonstrated in several instances. Treatment with FR resulted in reduced tumor growth in mouse xenografts,^19,57^ reduced glucose uptake in uveal melanoma cells in vitro and in vivo,^58^ and decreased colony formation of uveal melanoma cells in 3D cell culture.^59^ As these effects cannot be attributed to the GDI properties of FR, inhibitor binding was hypothesized to switch the nucleotide preference of Gα_q_^Q209L/P^ from GTP to GDP in an allosteric fashion.^17^ The additionally proposed “molecular adhesive” mechanism of action of YM and FR might provide an explanation for effector protein silencing downstream of constitutively active Gα_q_^Q209L/P^.^71^

## Application of YM and FR as Tool Compounds in Molecular Pharmacology

YM and FR possess several qualities of probe compounds:^60^ they are highly potent, showing a binding affinity in the low nanomolar to subnanomolar range,^24^ and a comparable inhibitory potency in calcium mobilization assays.^50^ In other assay readouts, such as dynamic mass redistribution assays or ERK phosphorylation assays, the potency has been found to be lower, but still in a satisfactory range (IC_50_ < 1 μM).^52^ Biophysical proof of target engagement has been demonstrated in several ways, most notably by X-ray crystallography of YM and FR in complex with the heterotrimeric, GDP-bound Gα_q/11_β_1_γ_2_ protein^23^ and by radioligand binding.^24^ Despite the unsaturated moiety in the dehydroalanine block of YM and FR that forms a potential Michael acceptor (commonly an α,β-unsaturated carbonyl group that can react with a nucleophile, forming a carbon–carbon bond), both compounds do not bind in a covalent fashion.^22^ However, no inactive analogue of those compounds is publicly available. Compounds such as YM-1 or FR-core, which share a major part of the structure and show greatly reduced affinity for the Gα_q_ protein, were only synthesized or purified at laboratory scale. Conversely, no potent but structurally unrelated Gα_q_ protein inhibitor is known to date.

Both molecules have been extensively characterized in cell-based assays in multiple readouts, including, but not limited to calcium mobilization, IP_1_ accumulation, platelet aggregation, serum response element reporter gene assays, and dynamic mass redistribution.^38,50,51,56^ Virtually any investigated readout based on Gα_q/11_ protein activity was found to be suitable to characterize the activity of YM and FR, and in turn, YM and FR can be used as tool compounds to block any of these pathways. Notably, the potency derived from different assay systems can vary to a large extent: calcium mobilization assays yielded IC_50_ values that corresponded fairly well to the affinity values determined in radioligand binding experiments,^50^ while IP_1_ accumulation assays yielded potencies that were approximately 1 order of magnitude lower,^37^ and label-free dynamic mass redistribution assays potencies were reduced by a further order of magnitude.^52^ Thus, the concentration of YM and FR required for full signal suppression is highly dependent on the employed assay system, and if possible, a concentration-response curve should be recorded prior to performing experiments in a yet uncharacterized readout to find the optimal working concentration for YM or FR. Several published potencies in different assay systems for YM and FR are listed in Table 1.

In a laboratory setting, YM and FR are well-usable compounds that show neither significant solubility nor stability issues, display no off-target effects even at high concentrations, bind quickly, and are not directly cytotoxic. These properties are clearly advantageous compared to PTX, which usually requires at least 4 h of incubation time or cotransfection of a constitutive PTX expression plasmid. For pilot experiments in cell-based readouts, a Gα_q/11_ inhibitor concentration of 100 nM and a preincubation time of 30 min prior to intervention or measurement is a good starting point. For most setups, this concentration will be sufficient as it is approximately 10 times higher than the apparent affinity of the drugs to the Gα_q_ protein and will therefore inhibit around 90% of the Gα_q/11_ proteins. In cases requiring full inhibition of Gα_q/11_ proteins, an inhibitor concentration of 1 μM may be desirable. Higher inhibitor concentrations, such as 10 μM or even 1 mM, are typically not required, nevertheless off-target effects are not expected even for very high concentrations (except for a putative partial inhibition of the Gα_15_ protein,^61^ in the case it is expressed in the investigated system).^52,56^ Membrane permeation of YM and FR does not pose a problem, exemplified by YM- and FR radiotracer binding to intact platelets with similar association kinetics.^24^ A thirty-minute incubation prior to stimulation of a Gα_q/11_-mediated signal exceeds the association half-life of the compound by far and should therefore be sufficient for the inhibitor to bind at near-equilibrium conditions.^62^ Longer inhibitor exposure, e.g., in overnight experiments, is not expected to cause cytotoxic side effects as demonstrated by the application of FR in serum response element assays with a total FR incubation time of 7 h or more.^19,56^ Washing out YM is possible due to its shorter residence time at the Gα_q_ protein (but can require several washing steps with in-between incubation periods), while FR binds in a pseudoirreversible and thus wash out-resistant manner.^22,24^

While no other similarly potent and selective compounds binding to other Gα protein families are available, inhibition of two other Gα proteins, Gα_s_ and Gα_15_, by YM and FR was unlocked via reverse mutation of the respective Gα protein’s “inhibitor binding site” to the Gα_q_ inhibitor binding site.^52,63^ This allows pharmacological inhibition of Gα_s_ and Gα_15_-mediated signaling pathways in cellulo, but requires previous knockout of the respective native protein and will simultaneously lead to Gα_q/11_ protein inhibition (unless these proteins are knocked out as well and rescued by inhibitor-resistant Gα_q_ variants). While suitable genome-edited human embryonic kidney 293 cell lines have been described,^64^ the translation of results to more native systems is difficult as pharmacological modulation of said Gα proteins is not possible outside of genome-edited, transfected cells.

YM and FR have been used as tool compounds in basic research and translational science on several occasions, with YM yielding 133 results and FR yielding 65 results in a PubMed keyword search (04.07.2023), with an increasing interest in YM after its commercialization, and in FR following its first comprehensive pharmacological characterization.^17^ Application of YM and FR as tool compounds in cellulo has significantly improved our current model of understanding G protein signaling. For instance, Pfeil et al. have characterized Gα_q_ as a master switch for Gα_i_βγ-mediated calcium release, by demonstrating that pharmacological inhibition by FR or genetic ablation of Gα_q_ proteins also abolished Gα_i_βγ-mediated calcium mobilization.^65^ Similarly, White et al. demonstrated that Gα_q/11_ inhibition reduced the duration of parathyroid hormone receptor-mediated cAMP production, providing insight in the spatiotemporal regulation of Gα_s_-driven signaling processes.^66^ Pharmacological inhibition of Gα_q/11_ signaling may also be advantageous in obesity, as Gα_q/11_ activation in adipose tissue leads to decreased energy expenditure and decreased uncoupling-protein 1 expression, and a decreased frequency of brown adipocytes.^67^ From a translational perspective, Onken et al. and Annala et al. independently demonstrated that FR can target constitutively active Gα_q_ mutants driving uveal melanoma, thereby inhibiting oncogenic properties of constituvite Gα_q/11_ signaling, while having no effect on non-Gα_q_-mediated malignancies, opening up a possible future for Gα_q/11_ inhibition as a therapeutic mechanism to treat uveal melanoma.^19,57^

## Employing YM and FR in Animal Studies

Besides using YM and FR in classical in cellulo or in vitro systems, inhibition of Gα_q/11_-mediated signaling in more complex systems and translational models is of high interest to disentangle the contribution of this signaling pathway in the context of various physiological and pathological conditions. However, the list of studies performed in vivo is rather short, despite their promising outcomes, and revolves around the investigation of *asthma bronchiale*, pain, thrombosis, and uveal melanoma.^19,45,68^ Importantly, Gα_q_ proteins in rat and mouse are identical to the human Gα_q_ protein with the exception of one amino acid in the helical domain (I91 V from human to mouse/rat). Consequentially, they share a virtually identical affinity for both YM and FR, confirmed by radioligand binding assays.^24^ Due to low predicted oral bioavailability, all animal studies performed with YM or FR to this date used intratracheal, intraperitoneal, or intrathecal application routes. The two most comprehensive animal studies focus on the bronchiodilating effect of FR in a model of asthma bronchiale^45^ and on antinociceptive effects exerted by YM.^68^

Matthey et al. showed that inhalation of 2.5 μg FR resulted in strong and long-lasting antiasthmatic effects of FR outperforming those of conventionally used antiasthmatic drugs, such as the β_2_-adrenoceptor agonist salmeterol, without inducing systemic side effects. Additionally, FR protected mice from allergen-induced airway hyperreactivity.^45^

Studying the effect of Gα_q/11_ signaling on pain transmission, Marwari et al. detected long-lasting antinociceptive effects by intrathecal application of YM, which intriguingly displayed a strong synergy when applied in combination with morphine by reducing the excitability of dorsal root ganglia.^68^

Due to the broad impact of Gα_q_ signaling as a major signal transduction pathway downstream of GPCRs, systemic application of Gα_q_ protein inhibitors was initially expected to be highly toxic at relevant concentrations. After the initial isolation of YM, Kawasaki et al. noticed that the compound displayed antithrombotic effects, but also decreased the heart pressure in anaesthetized rats and dogs at doses of 30 μg kg^–1^, administered as bolus injection.^69^ In agreement with this observation, Matthey et al. observed hypotensive effects in mice following the injection of 12.5 μg FR per animal into the jugular vein.^45^ At a dose of 2.5 μg FR per animal administered in the same fashion, no noticeable side effects were observed. A possible explanation for the lack of side effects observed by Matthey et al. might come from the preferred enrichment of the drug molecule in lung tissue detected in the same study by an LC/MS-MS protocol.^45^

Upon subcutaneous administration of 500 μg kg^–1^ YM, Marwari et al. observed reduced locomotion in mice. Local administration of YM by intrathecal injection, however, caused no side effects.^68^ This observation has not been reported by other studies so far. A further study investigated growth inhibition of uveal melanoma xenograft in mice, and administered 10 μg of FR by intraperitoneal injection, but did not report on the observation of any side effects.^19^

Thus, the limited information available from published studies does not allow a comprehensive assessment of side effects from systemic Gα_q/11_ inhibition. However, published reports seem to be conflicting, which may be caused by differences between species, strains, and administration routes. For instance, in one case, administration of 30 μg kg^–1^ YM (i*.*v. bolus injection) caused a significant drop in blood pressure in rats and dogs,^69^ while in another case, no blood pressure drop was reported despite using a more than 10-fold dose of YM (s*.*c. injection in mice).^68^ In the third case, a dose of 12.5 μg FR per mouse (i*.*v. bolus injection, corresponding to approximately 400 μg kg^–1^) was required to elicit a drop of blood pressure, which was not noticed at a lower dose of 2.5 μg per animal (approximately 80 μg kg^–1^).

Most importantly, the previously mentioned animal studies suggest that therapeutic application of FR and YM is indeed possible. Nevertheless, the route of administration and/or the dosage require careful consideration, depending on the investigated conditions, to eliminate toxic side effects while reaching suitable drug concentrations in the compartment of interest. Generally, high systemic exposure to the Gα_q_ protein inhibitors should be limited if possible as shown by reports of inhibited locomotion and reduction of blood pressure observed in aforementioned studies. Compared to their widespread application in molecular pharmacology, the under-use of Gα_q/11_ protein inhibitors in vivo may stem in one part from the relatively high amount of expensive compound that needs to be purchased for such experiments, and in other parts from a concern regarding adverse drug effects from systemic Gα_q/11_ inhibition. Due to the higher metabolization rate of FR, it may be advisible to use YM to inhibit Gα_q/11_ proteins in vivo. A possible future development of a locally targeted YM/FR-therapy, either by chemical modification or a sophisticated delivery system, will greatly facilitate the investigation of the Gα_q/11_ signaling contribution to further pathologies in vivo by decreasing the risk of systemic side effects at elevated drug concentrations, and may even constitute a much-needed treatment for uveal melanoma patients.

## Summary and Recommendations

The natural products YM and FR are potent and selective inhibitors of Gα_q_, Gα_11_, and Gα_14_ proteins and are currently the only potent and commercially available nonprotein inhibitors of heterotrimeric G proteins. They both fulfill most of the criteria defined for probe compounds, making them ideally suited for the investigation of Gα_q/11_-mediated processes. In contrast to protocols for PTX and CTX, which involve either overnight incubation, cell permeabilization, or transfection of an expression vector, the application of YM and FR is straightforward, and thus they have become well-accepted and frequently used tool compounds in molecular pharmacology. In combination with genetic ablation of Gα_q/11_ proteins or with Gα protein-specific biosensors, YM and FR add to a powerful toolbox to unambiguously illuminate processes such as GPCR-Gα coupling or the role of Gα_q/11_ proteins in signal transduction processes,^9,65,67^ and their potential can be extended to engineered inhibitor-sensitive Gα_s_ and Gα_15/16_ proteins.^52,63^ Both compounds can be recommended without any further restrictions for most in vitro applications. For experiments which include washing steps, the usage of FR may be preferred due to its pseudoirreversible target binding. In contrast, application of YM over FR may be preferable for in vivo experiments due to its slower metabolization rate.

A recent and noteworthy publication reported two peptide-based, state-selective modulators of the Gα_s_ protein, unlocking pharmacological modulation of the Gα_s_ protein and further proving the druggability of Gα proteins by cyclic peptides. However, the published compounds have not been employed as tool compounds outside of the original study so far and are not commercially available.^70^ While new chemotypes unlocking the inhibition of other wild-type Gα proteins or targeted YM/FR-delivery systems to specific tissues are highly desirable for basic and translational research, the promising and sometimes also surprising outcome of experiments with YM and FR encourages the further investigation of Gα_q/11_ proteins in complex systems and pathways. This is not limited to cellular signal transduction readouts or animal studies, but also extends to in vitro systems consisting of primary cells or tissues, organoids, or organ-on-a-chip models, which are less amenable to genetic manipulation of any sort compared to cancer cell cultures. Especially in the investigation of complex systems, the Gα_q/11_ signaling pathway may still hold a few surprises that can be uncovered by the application of YM and FR to further shape the understanding of G protein-mediated signaling processes.

## Abbreviations

Ac: acetyl
ADP: adenosine diphosphate
BGC: biosynthetic gene cluster
BRET: bioluminescence energy resonance transfer
CTX: cholera toxin
CYP: cytochrome P 450
DAG: diacylglycerol
DAG: diacylglycerol
Dha: dehydroalanine
ERK: extracellular signal-regulated kinase
FR: FR900359
G protein: Heterotrimeric guanine nucleotide-binding protein
GDI: guanine nucleotide dissociation inhibitor
GDP: guanosine diphosphate
GPCR: G protein-coupled receptor
GTP: guanosine triphosphate
Hle: hydroxyleucine
IP_1/3_: inositolmono/trisphosphate
LC-MS/MS: liquid chromatography - tandem mass spectrometry
Pla: phenyllactic acid
PLC-β: phospholipase C-β
Pp: propionyl
PTX: pertussis toxin
YM: YM-254890
